# Effects of monensin and a blend of magnesium oxide on performance, feeding behavior, and rumen morphometrics of Zebu beef cattle fed high-starch diets

**DOI:** 10.1093/tas/txae131

**Published:** 2024-09-13

**Authors:** Javier A Bethancourt-Garcia, Marcio M Ladeira, Karolina B Nascimento, Germán D Ramírez-Zamudio, Javier A Moreno Meneses, Matheus C Galvão, Thiago F Bernardes, Mateus P Gionbelli

**Affiliations:** Department of Animal Science – Universidade Federal de Lavras, Lavras, MG, Brazil; Department of Animal Science – Universidade Federal de Lavras, Lavras, MG, Brazil; Department of Animal Science – Universidade Federal de Lavras, Lavras, MG, Brazil; Department of Animal Science – Universidade Federal de Lavras, Lavras, MG, Brazil; Department of Medicine Veterinary and Animal Science, Universidad de Ciencias Aplicadas y Ambientales (UDCA), Cartagena, Bolivar, Colombia; Department of Animal Science – Universidade Federal de Lavras, Lavras, MG, Brazil; Department of Animal Science – Universidade Federal de Lavras, Lavras, MG, Brazil; Department of Animal Science – Universidade Federal de Lavras, Lavras, MG, Brazil

**Keywords:** alkalizing additives, antibiotic alternatives, ionophores, feedlot, magnesium

## Abstract

This study aimed to evaluate the effects of a blend of different sources of magnesium oxide associated or not with monensin, on productive, ruminal, and nutritional parameters of steers. Eighty-four Nellore steers with an initial body weight (BW) of 367.3 ± 37.9 kg were allocated to one of 28 pens, with three steers per pen. Each pen was considered an experimental unit. Using a completely randomized design with a 2 × 2 factorial arrangement, the following treatments were assigned to each pen: 1) Control (CON)—a basal diet without additive inclusion; 2) Magnesium oxide blend (MG)—basal diet plus a magnesium-based product (pHix-up, Timab Magnesium, Dinard, France) provided at 0.50% of dry matter (DM); 3) Monensin (MON)—basal diet plus 25 mg/ kg of DM of sodium monensin (Rumensin, Elanco Animal Health, Greenfield, IN); and 4) MG association with MON—basal diet plus MG + MON, at the same doses of the individual treatments. The experimental period lasted 100 d. Blood samples were collected on days 0, 13, and 70 to determine d-lactate levels. Daily feed intake was recorded, and animal ingestive behavior was visually observed on days 66 and 67. On day 70, skeletal muscle tissue samples were obtained through biopsy for gene expression analysis. At the end of the experimental period, carcass ultrasonography was conducted. Subsequently, the steers were slaughtered, and rumen epithelium samples were collected for morphometric analysis. The use of monensin, of magnesium oxide blend, and their interactions, were treated as fixed effects, while the pens were considered as a random effect. Statistical differences were considered when *P* < 0.05. Steers-fed MG-containing diets consumed approximately 0.6 kg more DM per day than those fed diets without this additive (*P* = 0.01; 11.3 vs. 11.9 kg/d). The inclusion of MG in the diet increased (*P* = 0.02) the average daily gain. There was a greater Longissimus muscle area (LMA) and LMA per 100 kg of BW (*P* ≤ 0.03) for steers-fed diets with MG. Steers-fed MON exhibited reduced mRNA expression of the Atrogin-1 and *mTOR* compared to steers-fed MG + MON diets (MON × MG: *P* ≤ 0.04). Steers-fed MON had 6.9% greater feed efficiency (*P* = 0.02). Papillae width was lesser for CON than other treatments (MON × MG: *P* = 0.02). In conclusion, the magnesium oxide blend improved performance and carcass traits in high-energy feedlot diets, while monensin enhanced feed efficiency, suggesting potential for their use as alternatives or complements in beef cattle nutrition.

## Teaser

Employing a magnesium oxide blend for finishing beef cattle enhances feed intake, animal performance, and carcass characteristics, making it a promising alternative for antibiotic additives.

## Introduction

Including high-starch content in feedlot diets has become an increasingly common practice to maximize growth performance and feed efficiency (Crawford et al., 2022). However, energy-dense diets achieved through high amounts of fast-fermenting carbohydrates can accumulate organic acids (Boerman et al., 2015). Consequently, the depression of ruminal pH increases the risk of metabolic disorders, such as ruminal acidosis and bloating, which in turn amplifies the inflammatory response due to damage to the rumen epithelium and cecum, ultimately affecting animal productivity (Owens et al., 1998; Nagaraja and Lechtenberg, 2007).

Adopting nutritional technologies is essential to effectively use high-energy diets and optimize nutrient absorption while reducing the risk of metabolic disorders (Tedeschi et al., 2003; Bach et al., 2018). Sodium monensin is widely used in Brazilian feedlots (Silvestre and Millen, 2021) and United States (Samuelson et al., 2016) due to its multiple benefits on rumen fermentation, animal performance, and feed efficiency (Duffield et al., 2012). However, public opinion opposes the use of antibiotics in animal nutrition, driven by the precautionary principle. In this sense, the rise of antibiotic-resistant bacterial strains in animal-derived products has prompted some countries to take measures against this issue (Russell and Houlihan, 2003; Cameron and McAllister, 2016).

Based on this, several products have been studied in ruminant nutrition to offer a viable and human-safe alternative to ionophores, such as magnesium oxide (Hashemi et al., 2014; Tsiplakou et al., 2017; Bach et al., 2018; Colombo et al., 2021; Nascimento et al., 2024a; Xiong et al., 2024), calcium oxide (Nuñez et al., 2014; Schroeder et al., 2014), calcium-magnesium carbonate, calcium-magnesium hydroxide (Arce-Cordero et al., 2021a, 2021b; Agustinho et al., 2022), and calcareous marine sources (Cruywagen et al., 2015; Neville et al., 2019). Among these alternatives, adding magnesium oxide to high-energy diets effectively prevents reductions in ruminal pH (Bach et al., 2018, 2023; Colombo et al., 2021; Nascimento et al., 2024). This supplementation also enhances dry matter (DM) and nutrient intake (Bach et al., 2018, 2023; Nascimento et al., 2024a), increases milk production (Bach et al., 2018), improves total-tract digestibility (Bach et al., 2023; Nascimento et al., 2024a; Xiong et al., 2024) and microbial crude protein synthesis (Nascimento et al., 2024a), alters the ruminal microbiome (Ramos et al., 2021; Bach et al., 2023), improve serum antioxidant capacity (Xiong et al., 2024), and reduces biomarkers of systemic inflammation (Colombo et al., 2021), with some effects being dose-dependent. Nevertheless, it is still necessary to further investigate the potential effects of using magnesium oxide, either alone or with monensin, on Zebu animals, which constitute the primary genetic resource for animal production in tropical regions.

Hence, this study aimed to assess the impact of a magnesium oxide blend associated or not with monensin on performance, feed intake, feeding behavior, carcass traits, and rumen epithelium morphometry of Nellore males fed starchy diets. We hypothesized that replacing sodium monensin with a magnesium-based product containing calcium oxide and other natural components would maintain an appropriate ruminal pH and yield beneficial effects on the performance, feeding patterns, and ruminal histological profile of steers, providing a safe and promising alternative for use in animal nutrition.

## Materials and Methods

### Animal Welfare

This study was performed in the feedlot of the Beef Cattle Experimentation Unit of the Universidade Federal de Lavras (Lavras, Minas Gerais, Brazil). All the procedures and handling of animals described were conducted following the guidelines of the Ethics Committee for Animal Experimentation of the Universidade Federal de Lavras (Protocol no. 043/2019).

### Experimental Management

Eighty-four immunocastrated Nellore steers with an initial body weight (BW) of 367.3 ± 37.9 kg were distributed into 28 pens (3 steers/pen), following a completely randomized design with a 2 × 2 factorial arrangement. There were seven pens per treatment, and each pen was considered an experimental unit. Each pen (4 m × 10 m) was equipped with an individual feeder and a collective water bin, which was shared between two adjacent pens. The experimental period extended over approximately 100 d. Before the beginning of the experimental period, steers were acclimated to the facilities. One day before the beginning of the experimental period, all animals received their initial immunization (Bopriva, Pfizer Animal Health, Parkville, Australia). The second immunization was administered 39 d after the first vaccination. Steers were also vaccinated against respiratory diseases (parainfluenza-3 virus and bovine respiratory syncytial virus) (Bovilis Bovigrip, MSD Animal Health, Rahway, London, UK), dewormed using ivermectin (Ivomec, Boehringer Ingelheim Animal Health do Brasil Ltda, São Paulo, Brazil) and properly identified.

At the beginning of the experimental period, the following treatments were randomly assigned to the pens: 1) Control (CON)—basal diet without additive inclusion; 2) magnesium oxide blend (MG)—basal diet plus a magnesium-based product (pHix-up, Timab Magnesium, Dinard, France) containing magnesium oxide (81%), calcium oxide (5.5%), as well as additional mineral components inherent in the original ores, which are extracted through various calcination processes conducted at temperatures ranging from 600 to 1,500 °C, which was provided at the level of 0.50% of DM; 3) monensin (MON)—basal diet plus 25 mg/ kg of DM of sodium monensin (Rumensin, Elanco Animal Health, Greenfield, IN); and 4) MG association with MON—basal diet plus MG + MON, at the same doses of the individual treatments. The basal diet consisted of corn silage, ground corn, rehydrated corn grain silage, soybean meal, urea, and a commercial mineral mixture (Table 1). The pHix-up product contained 82.4% magnesium oxide (MgO), 49.7% magnesium (Mg), and 4.26% calcium oxide (CaO). Since the basal diet was consistent across all treatments, any differences in magnesium and calcium levels in the diets were solely due to the inclusion of this product. The diet was formulated according to the Nutrient Requirements of Zebu and Crossbred Cattle—BR-CORTE 3.0 (Valadares Filho et al., 2016). Additionally, the animals were acclimated to the corn-based finishing diet over 13 d, following a step-up protocol. In the step-up protocol, the target finishing ration was limit-fed to 33.3%, 55.6%, and 77.8% of ad libitum intake from days 1 to 4, 5 to 8, and 9 to 13, respectively. During the finishing phase, the diet was provided ad libitum with 2 daily feedings, at 0700 hours (supply of 60% of the daily amount of diet) and at 0400 hours (supply of 40% of the daily amount of diet). The amount of diet offered was monitored to prevent the accumulation of leftovers exceeding 3%. Feed offered and refusals were weighed daily and sampled once a week for chemical analysis and adjustment of the dry matter intake (DMI). Samples were weighed, identified, and then dried at 55 °C in a forced ventilation oven for approximately 72 h. Subsequently, composite samples were separated into three periods (feedlot days 13 to 39, 40 to 70, and 71 to 100 of feedlot phase) and stored in a room temperature until analysis. Individual DMI was calculated based on the daily records of feed provided and the weight of refusals the following day, which was then divided among the three animals in each pen.

**Table 1.** Ingredients and chemical composition of experimental diets

Item	Treatments
*Ingredients, % of DM*	
Corn silage	23.0
Silage of rehydrated corn grain	10.9
Ground corn	52.1
Soybean meal	7.00
Soybean hulls	4.00
Urea	1.05
Ammonium Sulfate	0.11
Salt (NaCl)	0.35
Limestone	0.70
Dicalcium phosphate	0.30
Mineral nucleus^1^	0.035
Rumensin 200 (monensin)	0.01
pHix-up^2^	0.50
Inert material	0.51
*Chemical composition, % of DM*	
Dry matter	71.37
Metabolizable energy^3^, Mcal/kg DM	2.81
Net energy for maintenance, Mcal/kg	1.87
Net energy for gain, Mcal/kg	1.23
Total digestible nutrients	77.8
Neutral detergent fiber	20.0
Crude protein	13.3
Ether extract	3.41
Starch	52.5
Organic matter	95.3

^1^Mineral nucleus: inclusion level = 0.1% of the concentrate (1 kg per ton). Assurance levels per kilogram of product: 1.9 g I; 0.56 g Se; 1.50 g Co; 267.2 g Zn; 172 g Mn; 60 g Cu; 350 g Fe.

^2^pHix-up (Timab Magnesium, Dinard, France)—Magnesium oxide (MgO), magnesium (Mg), and calcium oxide (CaO) in the product equivalent to 82.4%; 49.7%, and 4.26%, respectively.

^3^ME = TDN (g/kg DM) × 4.4 × 0.82 (Carvalho et al., 2018).

### BW and Nutrient Accretion

The BW of the steers was recorded at the beginning and end of the adaptation period, and days 39, 70, and 100 of the experimental period using a digital balance (Toledo, model MGR 3000, Brazil). Weighing was conducted with a 16-h interval of water and feed restriction at the beginning of the adaptation period and at the end of the experimental period (days 1 and 100 of the feedlot phase). The average daily gain (ADG) of the steers for the entire period was determined by calculating the difference between the initial and final weights of the animals and then dividing that difference by the duration of the period.

At the end of the experimental period, ultrasonography was performed on the right side of each animal using an Aloka 500-V machine (Corometrics Medical Systems, Wallingford, CT) equipped with a 3.5-MHz, 17.2-cm linear array transducer. The following measurements were performed: longissimus muscle area (LMA, cm²), subcutaneous fat thickness of the longissimus muscle (SFT, mm), rump muscle length (cm), and rump fat thickness (cm). The images were captured as detailed by Meneses et al. (2024) and were then analyzed using ImageJ software (National Institutes of Health, Bethesda, Maryland, USA).

### Gene Expression Analysis in Skeletal Muscle

On day 70 of the experiment, a skeletal muscle biopsy was conducted for gene expression analysis. Tissue samples were collected from one animal per pen. To perform the biopsy, the lumbar region was shaved, and a subcutaneous local anesthetic (lidocaine hydrochloride HCl, 20 mg/mL, total volume 6 mL) was administered. The biopsy site was disinfected with iodophor (Laboratório Pinus, São Paulo, Brazil). Afterward, a one cm incision was made with a scalpel and a sterile Bergstrom biopsy needle was used to collect a sample (approximately 1 g) of longissimus thoracis muscle tissue from the 13th rib (on the right side). Later, an antibiotic spray was administered to the incision site. Continuous monitoring of all steers was conducted for 48 h following the biopsy.

Immediately after each biopsy, the muscle samples were placed in cryovials and swiftly frozen in liquid nitrogen. At the end of the biopsy procedure, samples were stored under −80 °C for subsequent gene expression analysis.

The RNA isolation, RNA integrity and quality assessment, cDNA synthesis, and qPCR analysis were performed as detailed by Nascimento et al. (2024b). The primer sequences (Table 2) were developed based on sequences available in the GenBank public database. The design process utilized the Oligo Perfect Design software (Primer Quest Tool). Subsequently, the primer sequences were analyzed through Oligo Analyzer 3.1 and Premier Bosoft software. Once the primer sequences were designed, they were procured through commercial synthesis (Invitrogen, Carlsbad, CA, USA). The relative expression levels were determined using the method outlined by Pfaffl (2001), which takes into account the CT values adjusted for the amplification efficiency specific to each primer pair.

**Table 2.** List of primers sets used to quantify mRNA expression of bovine genes by quantitative real-time PCR (RT-qPCR)

Gene name	Gene abbreviation	Access number	Primer	Function
Muscle ring finger 1	*MuRF1*	NM_001046155.1	**F** GGACAGATGAGGAAGAGGA **R** CCTCATCATCGCCTTACTGG	Protein degradation
Atrogin-1	*Atrogin-1*	NM_001046155.1	**F** CCTTGAAGACCAGCAAAACA **R** AGACTTGCCGACTCTTTGGA	Protein degradation
Ribosomal protein S6 kinase	*p7056k*	AY396564.1	**F** TTGAACCAAAAATCCGATCC **R** AGCACCTCTTCCCCAGAAA	Protein synthesis
Glycogen synthase kinase 3β	*GSK3B*	NM_001101310.1	**F** GCCCAGAACCACCTCCTTT **R** TGCTGCCATCTTTGTCTCTG	Protein synthesis
Eukaryotic translation initiation factor 4 E	*eIf4E*	NM_174310	**F** AAACCACCCCTACTCCGAAT **R** TGCCCATCTGTTCTGTAAAGG	Protein synthesis
Mammalian target of rapamycin	*mTOR*	XM_015475105.1	**F** GTCATGGAGGACACGGATTAG **R** GGACCAGTGAGGTAATGAGATG	Protein synthesis
Insulin-like growth factor 1 receptor	*IGFR1*	HQ703508.1	**F** TGCGGTTCTGTTGATAGTGG **R** TGGAGTGCTGTATGCCTCTG	Energetic metabolism
Actin beta	*β-Actin*	NM_173979.3	**F** GTCCACCTTCCAGCAGATGT **R** CAGTCCGCCTAGAAGCATTT	Endogenous control

### Feeding Behavior

Visual observation of feeding behavior was performed on days 66 and 67 of the experimental period. The feeding behavior evaluation involved continuous monitoring over a 48-h period, spanning both day and night, across two consecutive days. This monitoring was conducted by human observers, with a minimum of five observers per hour. To assess the activities of individual animals, each observer was responsible for evaluating a specific group of animals. Care was taken to position observers in a way that did not disrupt the natural behavior of the animals they were observing. During nighttime observations, artificial lighting within the facility was deliberately turned off, and flashlights were used to aid in the assessment of feeding behavior. The following activities were documented for each animal: the number of meals per day, the duration of time spent on eating, as well as the time dedicated to ruminating, and periods of idleness, all measured in minutes. These activities were monitored at 5-min intervals (throughout the entire 48-h evaluation period). Afterward, the duration of each activity was calculated in terms of minutes per day by dividing the obtained value by 2,880 (which corresponds to the total minutes in a span of 2 d). To determine the number of meals per day, the frequency with which each animal visited the trough for meals was monitored at each 5-min interval. Meal size was timed and recorded for each animal. To determine the animal behavior parameters of the pen, the averages obtained from the 3 animals that made up the pen were used.

### Particle Size and Sorting

Feed offered and orts samples were collected at days 0, 30, 45, 60, and 80 of the feedlot phase to determine particle-size distribution. To achieve this objective, the Penn State Particle Size Separator was employed, which consisted of screens with apertures of 19 mm (long), 8 mm (medium), and 4 mm (short), along with a fine pan. Briefly, the samples were weighed, and subsequently, on a flat surface, the sieves containing the sample were shaken horizontally in one direction 5 times, with the sieves being rotated 1/4 turn after each series of 5 shakes. Later, the material removed from each of the sieves and from the bottom pan was weighed again to determine the proportion retained in each of them (Heinrichs and Kononoff, 1996), determining the percentage of long, medium, short, and fine particles for each sample.

Feed sorting was determined by assessing the real intake of each fraction of the Penn State Particle Separator in relation to the predicted consumption of that fraction, following the method outlined by Leonardi and Armentano (2003). The actual intake of each specific fraction was computed as the disparity between the quantity of that fraction in the provided diet and the quantity in the orts. In contrast, the expected intake for each individual fraction was computed by multiplying the intake of the total diet by the proportion of that fraction within the offered diet. Values reaching 100% indicate no sorting, values below 100% suggest selective refusals (sorting against), while values exceeding 100% imply preferential consumption (sorting for) (Devries et al., 2014).

### Fecal pH and Starch

Fresh fecal samples were collected in the morning (0700 hours) using the spot technique from each animal on days 35 and 69 of the feedlot phase for the determination of fecal pH and starch concentration. Samples from the three animals in each pen were proportionally combined to create a daily composite sample for that pen. The fecal pH was promptly determined. To achieve this, 50 g of feces was diluted in 100 mL of water and mixed thoroughly using a stirring rod. Subsequently, fecal pH was measured using a previously calibrated portable pH meter (model HI 208; SP Labor, São Paulo, Brazil). The remaining fecal content was stored at −20 °C and later utilized for starch analyses.

### Chemical Analysis of Feedstuffs and Fecal Samples

All chemical analyses were conducted at the Animal Nutrition Laboratory within the Department of Animal Science at the Universidade Federal de Lavras (UFLA). Prior to analysis, the feed ingredients, refusals, and fecal samples were submitted to a drying process in a forced-air oven at 65 °C until a consistent weight was achieved. Subsequently, these samples were finely ground using 1 mm sieve through a Wiley mill (Wiley TE-680, Philadelphia, PA, USA) and then stored at ambient temperature. Moisture content (method No G-003/1), ash content (M-001/1), nitrogen content (CP; N-001/1), ether extract (EE; G-004/1), and ash- and protein-free neutral detergent fiber (NDFap; N-002/1), were determined in accordance with the analytical protocols outlined by the Brazilian National Institute of Science and Technology (INCT-CA) guidelines (Detmann et al., 2012). Non-fibrous carbohydrates (NFC) were calculated using the formula proposed by Detmann and Valadares Filho (2010): NFC = [100 – (% CP + % NDFap + % EE + % ash)]. Starch analysis was also conducted following the recommended INCT-CA procedures (method No G-007/1). Measurements were taken using a UV/visible spectrophotometer set to 630 nm.

### Slaughter and Carcass Assessment

At the end of the experimental period, all steers were slaughtered on the same day at a commercial slaughterhouse in Campo Belo (MG, Brazil), which was situated 57 km away from the experimental feedlot. All procedures conducted within the slaughterhouse adhered to humane slaughter practices and complied with the Sanitary and Industrial Inspection Regulation for Animal Origin Products (Brasil, 1997). The animals were rendered unconscious using the captive bolt technique, and subsequently, they underwent bleeding and dressing procedures. The hot carcass weight (HCW) was recorded after removing the head, hide, feet, and viscera. Following the HCW recording, the carcasses were stored in a refrigerated room at 4 °C for 24 h. The carcass yield was then calculated by dividing the HCW by the final BW and multiplying the result by 100.

### Ruminal Histological Profile

During the slaughter process, 1 cm^2^ samples of rumen tissue (from 2 steers per pen) were collected from the cranial–ventral sac and immediately immersed in a buffered 10% formalin solution for 48 h to assess papillae morphology. The rumen tissue samples were submitted to dehydration through a series of alcohol solutions with concentrations of 70%, 80%, 85%, 90%, 95%, and 100% ethanol, followed by clarification using two rounds of xylene. The tissue was then embedded in paraffin blocks and sliced into 3 μm thick sections using a rotary microtome (model MRP-09, Lupetec, Cidade, SP, Brazil). Three slides per sample were generated and promptly stained with hematoxylin and eosin, following the method described by Pluske et al. (1996). Histological images were captured at 4 × magnification using a bright-field Olympus CX31 microscope equipped with an Olympus BX51 camera (Olympus Corp., Tokyo, Japan). Papillae derived from four images were analyzed utilizing ImageJ software (National Institutes of Health, Bethesda, Maryland, USA). Sampling and measurements of the rumen papillae were consistently performed by the same observer. The length and width of papillae, as well as the thickness of papillae epithelium and keratin thickness, were measured following the protocol by Lesmeister et al. (2004) with modifications suggested by Mirzaei-Alamouti et al. (2016).

### Statistical Analysis

The data were analyzed using a 2 × 2 factorial arrangement, considering the use of monensin, magnesium oxide blend, and their interactions as fixed effects. The pens were considered as random effects. Thus, to assess the impact of monensin inclusion in the diet, the average outcomes for diets containing monensin were compared with those that did not include it. Likewise, to evaluate the effect of the magnesium oxide blend, diets with magnesium were compared to those without the additive. In instances where interaction effects were statistically significant (*P* < 0.05), individual treatment comparisons were conducted. The following statistical model was employed:


$$\begin{array}{l} Yij\;=\mu +\;M_{i}+\;P_{j}+\;{\left(MP\right)}_{ij}+{\;\varepsilon \;}_{ij} \end{array}$$

Where *Y*_*ij*_ = observed measurement; μ = overall mean, *M*_*i*_ = fixed effect of monensin, *P*_*j*_ = fixed effect of magnesium oxide blend (pHix-up), *MP*_*ij*_ = interaction between the effects of *M* and *P*, ɛ_*ij*_* *= the random error associated with *Y*_*ij*_, following a normal distribution with mean 0 and variance σe^2^, expressed as ε_ij_ ~ N (0, σe^2^).

All measurements were subjected to analysis using PROC GLM (SAS Inst., Inc., Cary, NC). Initial shrunk body weight (SBW) served as a covariate for feedlot performance variables and carcass characteristics, while DMI was used as a covariate for plasma d-lactate concentration. Statistical significance was determined when *P* ≤ 0.05, and trends were discussed when 0.05 < *P* ≤ 0.10. One steer from the CON treatment experienced muscle trauma two days before slaughter. As a result, all carcass measurements for this animal at the slaughterhouse were excluded from the analyses.

## Results

### Feedlot Performance, Nutrient Accretion, and Carcass Measurements

There was no MON × MG interaction for performance data, ultrasound measurements, or carcass traits (*P* ≥ 0.19; Table 3). Animals fed diets containing MON had similar (*P* ≥ 0.21) body weight, muscle measurements, fat deposition, and carcass yield compared to those that did not receive MON. The inclusion of MG in the diet increased the ADG (*P *= 0.02) by approximately 9.4%, while the final SBW was 13.5 kg greater for steers-fed diets with the magnesium oxide blend (*P* = 0.02; 498 vs. 511.5 kg for steer-fed diets without or with MG, respectively). The use of MG in finishing diets promoted approximately 10% and 7.5% additional LMA and LMA per 100 kg of BW (*P* ≤ 0.03) compared to animals fed without this additive (Table 3). There was also a trend (*P* = 0.09) towards increased subcutaneous fat thickness due to the use of MG in the diet (2.94 vs. 3.55 mm for steer-fed diets without or with MG, respectively). Despite the absence of effects of MG use in the diet on carcass yield (*P* = 0.75), there was a trend (*P* = 0.08) towards an approximately 7% greater HCW for animals receiving MG in their diet (Table 3).

**Table 3.** Effects of monensin (MON) and magnesium oxide blend (MG) on performance, nutrient accretion, and carcass traits of steers-fed high-energy diets

Item	Treatments^1^				SEM	*P* value		
	CON	MON	MG	MON + MG		MON	MG	MON × MG
*Feedlot performance*								
Initial BW, kg	365	367	370	367	14.9	0.86	0.98	0.85
Initial SBW, kg	349	347	351	349	13.4	0.87	0.92	0.96
Final SBW, kg	493	503	511	512	5.58	0.33	0.02	0.39
ADG, kg/d	1.43	1.54	1.62	1.63	0.05	0.32	0.02	0.38
*Ultrasonic carcass measurements*								
LMA, cm^2^	79.6	86.3	91.4	91.2	2.69	0.24	<0.01	0.21
LMA/100 kg SBW, cm^2^	16.2	17.1	17.9	17.9	0.53	0.42	0.03	0.38
RML, cm	9.87	9.48	9.92	10.03	0.185	0.45	0.11	0.19
SFT, mm	3.04	2.83	3.73	3.36	0.358	0.43	0.09	0.82
Rump fat, mm	4.91	5.19	4.87	5.64	0.479	0.27	0.68	0.62
*Slaughterhouse carcass measurements*								
HCW, kg	270	276	278	282	3.83	0.21	0.08	0.95
Carcass yield, %	54.9	54.8	54.3	55.2	0.40	0.36	0.75	0.23

^1^Treatments: CON = diets without additives; MON = basal diet plus 25 mg/kg of DM of sodium monensin (Rumensin, Elanco Animal Health, Greenfield, IN); MG = basal diet plus a magnesium oxide blend product (pHix-up, Timab Magnesium, Dinard, France) provided at 0.50% of DM; MON + MG = basal diet plus MG + MON, at the same doses of the individual treatments.

### Gene expression in Skeletal Muscle

There was an interaction between MON and MG for the expression of the *Atrogin-1* and *mTOR* genes (*P* ≤ 0.04; Table 4). Animals receiving MON exhibited reduced mRNA expression of the *Atrogin-1* and *mTOR* compared to steers-fed diets containing MG + MON. No effects of MON or MG inclusion were observed on the other evaluated genes (*P* ≥ 0.11).

**Table 4.** Effects of monensin (MON) and magnesium oxide blend (MG) on relative gene expression in the longissimus thoracis muscles of steers-fed high-energy diets

Item	Treatment				SEM	*P* value		
	CON	MON	MG	MON + MG		MON	MG	MON × MG
*MURF1*	1.00	0.76	0.88	0.83	0.13	0.32	0.86	0.48
*Atrogin-1*	1.00^ab^	0.67^b^	0.91^ab^	1.28^a^	0.15	0.86	0.11	0.04
*mTOR*	1.00^ab^	0.74^b^	0.99^ab^	1.04^a^	0.05	0.11	0.03	0.03
*P7056K*	1.00	0.78	0.89	0.72	0.17	0.30	0.65	0.90
*GSK3B*	0.99	0.90	0.91	1.03	0.07	0.91	0.76	0.19
*EIF4E*	1.00	0.80	0.90	0.96	0.07	0.45	0.70	0.13
*IGFR1*	1.00	0.95	1.04	1.12	0.12	0.91	0.42	0.66

^a,b^Statistical differences between means are denoted by superscript letters.

### Feed intake, Ingestive Behavior, and Particle Sorting

Steers-fed MG diets ingested approximately 0.6 additional kg of DM per day than steers-fed diets without this additive (*P* = 0.01; 11.3 vs. 11.9 kg/d for steer fed diets without or with MG, respectively; Table 5). Additionally, the inclusion of MG resulted in an approximately 3.7% greater DMI per kg of SBW (*P* = 0.03). On the other hand, no effects of MG use in finishing diets on feed efficiency were observed (*P* = 0.20; Table 5). The inclusion of MON in the diet did not affect DMI (*P* = 0.18), but it tended (*P* = 0.10) to decrease the DMI per kg of SBW (2.59 vs. 2.57 for steers-fed diets without and with MON, respectively). In contrast, the inclusion of MON in the diet promoted approximately a 6.9-point increase in feed efficiency compared to diets without MON (*P* = 0.02; Table 5). A trend (*P* ≥ 0.09; Table 5) indicates reduced time spent in idleness for steers-fed diets with MON compared to those on other diets. However, no MON × MG interaction was found for other ingestive behavior parameters (*P* ≥ 0.11; Table 5). In general, incorporating MG into the diet did not significantly affect the animals’ feeding behavior pattern (*P *≥ 0.13). However, there was a tendency (*P* = 0.10) for steers-fed diets with MG to have a greater DMI per meal compared to those fed diets without MG. Animals fed diets containing MON spent 11% more time eating (*P* = 0.04; without MON = 170 vs. with MON = 190 min/d) than animals without MON in their diet. The time spent eating relative to DMI was also greater (*P *= 0.02) for animals receiving MON. There was a tendency for more meals per day (*P* = 0.09) and greater DMI per meal (*P* = 0.10) for steers-fed MON (Table 5). Although no effects of MG use or interaction between MON × MG on particle sorting were observed (*P *≥ 0.53), there was a greater selection for medium-sized particles (*P* = 0.05) and a tendency for greater selection for long particles (*P* = 0.08) in animals with MON inclusion in the diet.

**Table 5.** Effects of monensin (MON) and magnesium oxide blend (MG) on feeding intake, behavior, and particle sorting of steers-fed high-energy diets

Item	Treatments^1^				SEM	*P* value		
	CON	MON	MG	MON + MG		MON	MG	MON × MG
*Feed intake*								
DMI, kg/d	11.4	11.2	12.1	11.7	0.22	0.18	0.01	0.65
DMI, g of feed/kg of SBW	2.58	2.54	2.70	2.61	0.41	0.10	0.03	0.60
Feed efficiency, %	12.7	13.9	13.4	14.0	0.36	0.02	0.20	0.43
*Feeding behavior*								
Time spent idleness, min	931.97	865.51	915.93	927.21	22.29	0.22	0.31	0.09
Time spent ruminating, min	135.07	159.83	146.56	143.59	8.63	0.22	0.78	0.12
Time spent eating, min	168.89	195.05	171.59	184.06	9.13	0.04	0.66	0.47
Meal length, min	18.41	20.49	20.21	19.49	0.86	0.43	0.64	0.11
Meals per day, *n* (d)	8.02	8.62	7.48	8.45	0.34	0.09	0.49	0.68
DMI, kg of DM	12.20	11.98	12.55	12.42	0.49	0.72	0.43	0.93
DMI per meal, kg	1.58	1.44	1.68	1.56	0.55	0.10	0.10	0.94
Time spent eating/DMI, min/kg of DM	12.94	15.65	13.26	14.74	0.76	0.01	0.69	0.42
Time spent ruminating/DMI, min/kg of DM	11.69	13.47	11.10	11.37	0.85	0.24	0.13	0.39
*Particle sorting, arbitrary units*								
Long	1.027	1.033	1.026	1.033	0.001	0.08	0.86	0.86
Medium	1.024	1.029	1.022	1.030	0.001	0.05	0.83	0.53
Short	1.013	1.016	1.010	1.016	0.001	0.13	0.60	0.61
Fine	0.986	0.983	0.985	0.981	0.001	0.30	0.78	0.90

^1^Treatments: CON = diets without additives; MON = basal diet plus 25 mg/ kg of DM of sodium monensin (Rumensin, Elanco Animal Health, Greenfield, IN); MG = basal diet plus a magnesium oxide blend product (pHix-up, Timab Magnesium, Dinard, France) provided at 0.50% of dry matter; MON + MG = basal diet plus MG + MON, at the same doses of the individual treatments.

^a,b^Statistical differences between means are denoted by superscript letters.

### Concentration of Starch in Feces and Fecal pH

The inclusion of MON in finishing diets tended (*P* = 0.08; Table 6) to increase the starch concentration in feces by approximately 7.3%. At 35 d in the feedlot, there was a trend toward MON × MG (*P* = 0.09) use in the diets, with lesser fecal pH in the CON group compared to the other treatments. At 69 d in the feedlot, the fecal pH was not influenced by the nutritional plans studied (Table 6).

**Table 6.** Effects of monensin (MON) and magnesium oxide blend (MG) on starch concentration in feces and fecal pH of steers-fed high-energy diets

Item	Treatments^1^				SEM	*P* value		
	CON	MON	MG	MON + MG		MON	MG	MON × MG
*Starch concentration*								
Fecal starch, %	8.93	9.86	9.05	9.44	0.369	0.08	0.69	0.47
*Fecal pH*								
Day 35 of feedlot	5.80	6.00	6.00	5.95	0.072	0.32	0.30	0.09
Day 69 of feedlot	5.83	5.98	5.96	5.94	0.068	0.34	0.51	0.24

^1^Treatments: CON = diets without additives; MON = basal diet plus 25 mg/kg of DM of sodium monensin (Rumensin, Elanco Animal Health, Greenfield, IN); MG = basal diet plus a magnesium oxide blend product (pHix-up, Timab Magnesium, Dinard, France) provided at 0.50% of DM; MON + MG = basal diet plus MG + MON, at the same doses of the individual treatments.

^a,b^Statistical differences between means are denoted by superscript letters.

### Papillae Morphology

There was a MON × MG interaction for papillae width (*P* = 0.02). Monensin increased papillae width when there was no MgO but did not affect papillae width when MgO was in the diet (Table 7). Papillae surface area, papillae height, and keratinized layer thickness were not influenced by the treatments applied (*P *≥ 0.41; Table 7).

**Table 7.** Effects of monensin (MON) and magnesium oxide blend (MG) on ruminal papilla morphometric measurements of steers-fed high-energy diets

Item	Treatments^1^				SEM	*P* value		
	CON	MON	MG	MON + MG		MON	MG	MON × MG
Papillae surface area, cm^2^	0.74	0.79	0.86	0.83	0.05	0.88	0.17	0.44
Papillae width, mm	0.35^b^	0.44^a^	0.41^ab^	0.41^ab^	0.02	0.04	0.45	0.02
Papillae height, mm	1.68	1.89	1.93	1.89	0.16	0.63	0.46	0.44
Keratinatized layer thickness, μm	60.50	59.34	58.17	61.23	2.44	0.71	0.93	0.41

^1^Treatments: CON = diets without additives; MON = basal diet plus 25 mg/kg of DM of sodium monensin (Rumensin, Elanco Animal Health, Greenfield, IN); MG = basal diet plus a magnesium oxide blend product (pHix-up, Timab Magnesium, Dinard, France) provided at 0.50% of DM; MON + MG = basal diet plus MG + MON, at the same doses of the individual treatments.

^a,b^Statistical differences between means are denoted by superscript letters.

## Discussion

The current study investigated the potential use of a magnesium-based product containing calcium oxide and other natural components (as an additive in ruminant nutrition), hypothesizing that its use could benefit beef cattle’s performance. Consistent with our hypothesis, the use of the magnesium oxide blend was able to improve the ADG of steers-fed high-energy diets, which resulted in a greater BW at the end of the experimental period. Additionally, including MG in the diet increased the loin eye area of the steers and tended to improve HCW. Therefore, a consistent effect of its use on animal performance and nutrient accretion was observed.

Such an effect may be associated with several factors. The first of them is that the use of MG increased DMI. Therefore, the greater feed intake for the steers-fed magnesium oxide blend provided a greater supply of nutrients and energy for metabolic processes, including tissue synthesis, contributing to the increase in BW measurements. Additionally, it is important to highlight that magnesium acts as a significant intracellular cation, functioning as a vital cofactor in enzymatic reactions that are essential for all major metabolic pathways (Goff, 2018). It also plays a pivotal role in fundamental homeostatic processes, including glycolysis and energy-driven membrane transport, cyclic-AMP synthesis, and genetic information transmission (NASEM, 2016). Therefore, considering the importance of magnesium in metabolic functions, this may have reflected an improvement in overall organism functioning, consequently enhancing animal performance.

In line with our findings, Hashemi et al. (2014) observed that adding magnesium oxide to the diets of lambs fed a high-concentrate diet had beneficial effects. Specifically, the inclusion of 0.05% magnesium oxide in the diet increased DMI, which resulted in improved pre-slaughter weight, hot and chilled carcass weight, and dressing percentage. Another study by Xiong et al. (2024) evaluated the combined use of magnesium oxide and sodium bicarbonate (2.5 g/kg MgO + 7.5 g/kg NaHCO_3_ or 5 g/kg MgO + 7.5 g/kg NaHCO_3_) in feedlot lamb diets, comparing it to diets without additives or those containing only sodium bicarbonate (15 g/kg NaHCO_3_). The results showed that animals receiving buffers in their diets had greater ruminal pH and total VFA. Moreover, the combination of 2.5 g/kg MgO + 7.5 g/kg NaHCO_3_ led to greater ADG, final BW, ribeye area, and dressing percentage compared to the other groups, which aligns with our study. Furthermore, the presence of calcium oxide in the product tested in this study may have influenced the observed effects on animal performance. Supporting this, evidence from Nuñez et al. (2014) indicates that calcium oxide positively impacts ruminal pH, with doses up to 1.6% effectively enhancing the performance of feedlot cattle.

Despite the greater intake observed in animals fed MG in their diet, no substantial effects on ingestive behavior were observed. Only a tendency towards increased DMI per meal was observed in animals fed diets with MG inclusion, aligning with the overall greater DMI. Although it is beyond the scope of this study to evaluate the ruminal pH values based on the treatments used, the increased feed intake observed in steers-fed the MG blend may be associated with an improvement in the ruminal pH. Under a potential nutritional challenge scenario, such as under high-grain diets, there is organic acids accumulation in the rumen, including VFA and lactic acid, which reflects an imbalance between microbial production, microbial utilization, and ruminal absorption of organic acids (Nagaraja and Titgemeyer, 2007). As a result, this situation can cause a decline in fiber digestibility, elevate the concentration of VFAs (particularly propionate), and induce alterations in rumen osmolality, ultimately resulting in a decreased feed intake (Allen, 2000). In this sense, in a supplementary study performed by our lab (Nascimento et al., 2024a), the magnesium oxide blend was administered at varying doses (0, 2.5, or 5.0 g/kg of DM) to Nellore bulls following the same experimental diet as utilized in this study. It was observed that increasing MG supplementation levels in the diet had a linearly positive effect on ruminal pH, with the 5.0 g/kg of DM dosage (the same as tested in this trial) yielding the most favorable intake response. Additionally, other studies involving ruminants (Bach et al., 2018; Neville et al., 2019; Colombo et al., 2021) have demonstrated that magnesium-based products can improve ruminal pH stability, which supports the increased DMI observed in the present study.

The primary factors influencing the availability of magnesium are the potassium (K) concentration and the source of magnesium (Tebbe et al., 2018). Based on this, ionophores such as monensin have been proposed to substantially enhance Mg2 + digestion, as evidenced by their ability to reduce ruminal K + concentrations, which in turn indicates a potential reduction in the inhibitory effect of K + on Mg2 + transport (Martens et al., 2018). In this sense, monensin is a feed supplement employed to enhance feed efficiency by depleting intracellular potassium in cells and selectively modifying the microbial population within the rumen (Bergen and Bates, 1984). Nevertheless, this study confirmed that there is no substantial interaction between magnesium oxide and monensin in terms of their combined effects on the studied parameters for steer fed high-energy diets.

The inclusion of MON in the diet did not impact the BW, ADG, nutrient accretion (ultrasonic carcass measurements), or carcass parameters. Additionally, although substantial effects on genes associated with protein turnover in skeletal muscle tissue have not been observed, was verified that steers-fed diets containing MG + MON exhibited greater expression of the gene encoding *mammalian target of rapamycin* (*mTOR*) compared to those fed exclusively with MON. The mTOR pathway is a highly regulated intracellular signaling cascade that is essential for protein synthesis. It acts as a metabolic sensor, integrating several upstream signals related to nutritional status, nutrient availability, cellular growth factors, and stress (Lee et al., 2007). When stimulated, mTOR activates the phosphorylation of key targets to stimulate messenger RNA translation into proteins (Fonseca et al., 2014), resulting in increased protein synthesis. This response may have occurred due to the greater numerical feed intake of animals receiving MON + MG compared to those fed solely with MON. Additionally, steers-fed MON showed lesser mRNA expression of *Atrogin-1*, a gene associated with the protein degradation process, compared to those fed MON + MG. Therefore, collectively these findings suggest greater protein turnover in animals fed diets containing MON + MG compared to MON.

While no significant effects of MON use on animal DMI were detected, there was a trend indicating a reduction in DMI when expressed in kg of DM per kg of SBW in steers-fed MON. Furthermore, steers-fed MON had enhanced feed efficiency compared to those without MON in their diet. Monensin is well-known for its ability to reduce feed intake while simultaneously enhancing feed efficiency. In a meta-analysis conducted by Duffield et al. (2012), based on data from studies involving growing and finishing beef cattle, it was verified that MON led to a decrease in DMI and an improvement in both ADG and feed efficiency. Monensin has the potential to enhance the efficiency of feed utilization by reshaping rumen fermentation processes, consequently augmenting the energy available for productive purposes. Monensin alters the rumen fermentation dynamics by selectively inhibiting gram-positive bacteria. This alteration stimulates the proliferation of gram-negative bacteria, resulting in an elevated production of propionate in the rumen (Ogunade et al., 2018). Consequently, this heightened propionate production further amplifies hepatic gluconeogenesis. Monensin also reduces methane production by impeding the proliferation of bacteria responsible for providing substrates (hydrogen and formate) required for methanogenesis (Bergen and Bates, 1984). Therefore, this change in the ruminal environment leads to an improvement in the overall energy status of ruminant animals, which explains the favorable effect observed in feed efficiency due to MON use.

Dietary additives can modify feeding behavior, which can be associated with factors such as palatability, nutrient concentration, metabolic variables, pH levels, or osmolality in bodily fluids and organs (González et al., 2012). These changes can act as feedback signals that impact the onset and the end of meals through the central nervous system. In this sense, monensin is well-known to promote an increase in propionate flow for gluconeogenesis in the liver, which, in turn, sends feedback signals to the brain, triggering behavioral mechanisms to end the meal (Allen et al., 2009). Consequently, it can induce a hypophagic effect, resulting in smaller meals and reduced daily feed intake (Allen et al., 2005). Therefore, the feeding behavior of monensin-supplemented animals was characterized by more frequent and smaller meals, consumed at a slower rate compared to animals fed diets without MON (Erickson et al., 2003). This condition led to more stable ruminal fermentation patterns throughout the day, reduced variability, and a smaller postprandial drop in ruminal fluid pH in monensin-fed animals (González et al., 2012). In the present study, steers-fed MON diets exhibited increased eating time compared to those without MON, spent more time eating relative to DMI, and tended to have more daily meals and consume larger amounts per meal. Additionally, animals fed MON showed a tendency for reduced idleness time compared to those on other diets. Hence, these results align only partially with the findings reported in the scientific literature regarding the use of MON on ingestive behavior. Additionally, there was also a greater selection for medium-sized particles and a tendency for greater selection for long particles in animals with MON inclusion in the diet. Thus, this mechanism may have occurred as an alternative to avoid metabolic disturbances in an attempt to increase physically effective fiber intake, to stimulate chewing activity, saliva flow, and ruminal pH (Rigueiro et al., 2023). Conversely, in a study examining the effects of MON, both alone and in combination with virginiamycin or zinc bacitracin on feedlot young bulls, Junior et al. (2023) also found that animals fed with MON displayed a preference for consuming larger and medium particles (>19 and >8 mm, respectively).

The ruminal epithelium plays a pivotal role in the uptake of short-chain fatty acids (SCFA) via the layered squamous rumen lining. Hence, in response to a challenging nutritional scenario (e.g., diets high in concentrates), the papillae projecting from the rumen wall may enlarge to maximize the surface area for SCFA absorption (Steele et al., 2011). This phenomenon is primarily governed by the action of butyrate, which, in turn, promotes cellular proliferation while inhibiting apoptosis (Sakata and Tamate, 1978; Mentschel et al., 2001). In addition to butyrate, propionate also seems to act as a stimulator of papillary growth (Vair et al., 1960). Nonetheless, if the normal rumen fermentation process is disturbed, the SCFA and lactate accumulation within the rumen may result in damage to the rumen epithelium. In this context, an excessive increase in cellular proliferation and abnormal cellular differentiation may manifest within the stratified squamous epithelium of the rumen wall, resulting in the development of parakeratosis (Steele et al., 2012). Additionally, this impairment can compromise the protective role of the ruminal epithelium, potentially allowing the passage of pathogenic substances from the rumen into the bloodstream (Monteiro and Faciola, 2020). In the current study, no effects of the use of MG on papilla morphology were observed. This suggests that this dietary additive did not induce significant histological changes that could either enhance or impair the absorption of acids by the rumen epithelium. On the other hand, steers-fed MON exhibited increased papilla width compared to the CON group, which may possibly be related to greater stimulation via propionate on ruminal epithelium by MON. In line with this, Pereira et al. (2014) observed alterations in the histology of rumen papillae in feedlot Nellore cattle when using MON. These responses were dose-dependent, with doses of 9 or 36 ppm of MON resulting in improved development of the rumen epithelium.

In addition, MON-fed steers had a greater starch concentration in their feces. This finding contradicted expectations, as previous research with steers indicated that monensin reduces starch digestion in the rumen while increasing starch digestion in the intestine (Muntifering et al., 1981). It is also plausible that the observed alterations in ingestive behavior, in response to potential changes in rumen acid–base balance, could significantly augment the outflow of starch to later segments of the gastrointestinal tract (Zinn et al., 1998; Swanson et al., 2002). Although a greater amount of starch was detected in the feces, no difference in fecal pH was observed, suggesting that fermentation conditions in the postruminal section remained unaltered (Owens et al., 1998; Erickson et al., 2003; González et al., 2012).

Additionally, there was a trend toward a MON × MG interaction for fecal pH, in which steers-fed CON diets tended to present a lesser value compared to animals fed diets with additives. These findings suggest that the absence of additives in the control group’s diet may have resulted in a greater escape of volatile fatty acids to the posterior digestive tract, impacting fecal pH. Therefore, this underscores the importance of including additives in energy-rich diets for ruminants.

## Conclusions

The magnesium oxide blend in high-concentrate diets improved feedlot performance and carcass characteristics, while monensin enhanced feed efficiency. There were no substantial effects on intake, ingestive behavior, or papilla morphology due to the combined use of monensin and magnesium oxide blend. These findings suggest potential for their use as alternatives or complements in beef cattle nutrition.

## Abbreviations

ADG: average daily gain
BW: body weight
DMI: dry matter intake
eIf4E: eukaryotic translation initiation factor 4 E
G:F: feed efficiency
GSK3B: glycogen synthase kinase 3β
HWC: hot carcass weight
LMA: *longissimus* muscle area
mTOR: mammalian target of rapamycin
MuRF1: muscle ring finger 1
p7056k: ribosomal protein S6 kinase
RML: rump muscle length
SBW: initial body weight obtained in fasting
SFT: subcutaneous fat thickness
